# *Bordetella pertussis* Strains with Increased Toxin Production Associated with Pertussis Resurgence

**DOI:** 10.3201/eid1508.081511

**Published:** 2009-08

**Authors:** Frits R. Mooi, Inge H.M. van Loo, Marjolein van Gent, Qiushui He, Marieke J. Bart, Kees J. Heuvelman, Sabine C. de Greeff, Dimitri Diavatopoulos, Peter Teunis, Nico Nagelkerke, Jussi Mertsola

**Affiliations:** National Institute for Public Health and the Environment, Bilthoven, the Netherlands (F.R. Mooi, M. van Gent, M.J. Bart, K.J. Heuvelman, S.C. de Greeff, D. Diavatopoulos, P. Teunis); Maastricht University Hospital, Maastricht, the Netherlands (I.H.M. van Loo); National Public Health Institute, Turku, Finland (Q. He); United Arab Emirates University, Al Ain, United Arab Emirates (N. Nagelkerke); University of Turku, Turku (J. Mertsola)

**Keywords:** Gram-negative bacterial infections, bacteria, respiratory tract infections, pertussis, whooping cough, pertussis, resurgence, vaccination, the Netherlands, research

## Abstract

Before childhood vaccination was introduced in the 1940s, pertussis was a major cause of infant death worldwide. Widespread vaccination of children succeeded in reducing illness and death. In the 1990s, a resurgence of pertussis was observed in a number of countries with highly vaccinated populations, and pertussis has become the most prevalent vaccine-preventable disease in industrialized countries. We present evidence that in the Netherlands the dramatic increase in pertussis is temporally associated with the emergence of *Bordetella pertussis* strains carrying a novel allele for the pertussis toxin promoter, which confers increased pertussis toxin (Ptx) production. Epidemiologic data suggest that these strains are more virulent in humans. We discuss changes in the ecology of *B. pertussis* that may have driven this adaptation. Our results underline the importance of Ptx in transmission, suggest that vaccination may select for increased virulence, and indicate ways to control pertussis more effectively.

## Abstract 2

A more virulent strain of the disease is emerging.

*Bordetella pertussis* causes whooping cough or pertussis, a respiratory disease that is most severe in infants. Before childhood vaccination was introduced in the 1950s, pertussis was a major cause of infant deaths worldwide. Widespread vaccination of children reduced the incidence of illness and deaths caused by pertussis (*1*). However, globally pertussis remains 1 of the top 10 causes of death in children (*2*). Further, in the 1990s a resurgence of pertussis was observed in several countries with highly vaccinated populations (*3*,*4*), and pertussis has become the most prevalent vaccine-preventable disease in industrialized countries. In the Netherlands, the estimated incidence of infection was 6.6% per year for the 3–79-year age group from 1995 through 1996 (*5*). Similar percentages have been found in the United States (*6*). One of the hallmarks of the pertussis resurgence is a shift in disease prevalence toward older persons who have waning vaccine-induced immunity (*7*).

The reemergence of pertussis has been attributed to various factors, including increased awareness, improved diagnostics, decreased vaccination coverage, suboptimal vaccines, waning vaccine-induced immunity, and pathogen adaptation. The relative contribution of these factors may differ between countries and is the subject of ongoing debate. Pathogen adaptation is supported by several observations. We and others have shown that antigenic divergence has occurred between vaccine strains and clinical isolates with respect to surface proteins, which confer protective immunity: pertussis toxin (Ptx), pertactin (Prn), and fimbriae (*8*,*9*). Strain variation was shown to affect vaccine efficacy in a mouse model (*10*–*13*). Because adaptation may involve the structure of virulence factors (by antigenic variation) and their regulation, we extended our studies on the evolution of *B. pertussis* by investigating polymorphism in the promoter of Ptx (*ptxP*), a major virulence factor and component of all pertussis vaccines (*1*). We provide evidence that expansion of strains with increased Ptx production has contributed to the resurgence of pertussis in the Netherlands.

## Methods

### Pertussis Notifications

Pertussis became a notifiable disease in the Netherlands in 1976. Notifications are submitted online by local health authorities. Other notifiable diseases are also monitored through this system, which falls under the responsibility of the Dutch National Institute of Health and Environment (*3*).

### Bacterial Strains

*B. pertussis* strains examined were obtained from 1935 through 2004. A total of 1,566 isolates, 879 from the Netherlands and 687 from other countries, were analyzed for polymorphism in *ptxP* (Technical Appendix). Eight strains isolated from patients in the Netherlands from 1999 through 2001 were selected to study Ptx and Prn production: B1834 (*ptxP1*), B1868 (*ptxP1*), B1878 (*ptxP1*), B1920 (*ptxP1*), B1836 (*ptxP3*), B1865 (*ptxP3*), B1917 (*ptxP3*), and B2030 (*ptxP3*) (Table 1).

**Table 1:** Characteristics of strains used for Ptx and Prn production experiments*

Strain	Year of isolation	Patient age, mo	*ptxP* allele	*prn* allele	*ptxA* allele
B1834	1999	28	PtxP1	Prn2	PtxA1
B1836	1999	3	PtxP3	Prn2	PtxA1
B1865	2000	2	PtxP3	Prn2	PtxA1
B1868	2000	35	PtxP1	Prn2	PtxA1
B1878	2000	45	PtxP1	Prn2	PtxA1
B1917	2000	44	PtxP3	Prn2	PtxA1
B1920	2000	9	PtxP1	Prn2	PtxA1
B2030	2001	3	PtxP3	Prn2	PtxA1

*Ptx, pertussin toxin; Prn, pertactin; *ptxP*, pertussin toxin promoter; *prn*, gene for pertactin; *ptxA,* gene for the A subunit of pertussin toxin.

### Sequencing

The primers 5′-AATCGTCCTGCTCAACCGCC-3′ and 5′-GGTATACGGTGGCGGGAGGA-3′ were used for amplification and sequencing of *ptxP* and correspond, respectively, to bases 60–79 and 633–614 of the *ptx* sequence with GenBank accession no. M14378. The *ptx* gene cluster from the strains B1834 (*ptxP1*), B1920 (*ptxP1*), B1917 (*ptxP3*), and B1831 (*ptxP3*) was sequenced completely. The sequences of the *ptx* gene clusters from strains B1834, B1920, B1917, and B1831 can be found under the following accession numbers, respectively: FN252334, FN252335, FN252336, and FN252333. The *ptxP1-ptxP11* sequences have been assigned accession nos. FN252323, FN252322, FN252324, FN252325, FN252326, FN252327, FN252328, FN252329, FN252330, FN252331, and FN252332.

### Pertussis Toxin and Pertactin Production

*B. pertussis* strains were grown on Bordet-Gengou agar plates supplemented with 15% sheep blood and incubated for 3 days at 35°C. Cells were harvested and suspended in 2 mL Verwey medium (*14*) per plate. Cells from 1 mL were collected by centrifugation and resuspended in Verwey medium to a concentration of 5 × 10^6^ bacteria/mL. Subsequently, 100 µL of this suspension (5 × 10^5^ CFU) was plated on Bordet-Gengou agar plates. After an incubation of 48 to 60 hours at 35°C, cells were harvested in 2.5 mL Verwey medium. The cell suspension was heat-inactivated for 30 min at 56°C and stored at 4°C. An ELISA was used to quantify Ptx and Prn. For Ptx, Maxisorp 96-well plates (Nunc International, Rochester, NY, USA) were coated with 100 µL of 0.04 mg/mL fetuin (Sigma-Aldrich, St. Louis, MO, USA) in 0.04 M carbonate buffer, pH 9.6, overnight at 4°C. For Prn, polystyrene 96-well plates (Immunolon II; Dynatech, Chantilly, VA, USA) were coated with 100 µL of a 2,000-fold dilution of polyclonal rabbit anti-Prn immunoglobulin (Ig)G (*15*) in 0.04 M carbonate buffer, pH 9.6, overnight at 20°C. Plates were blocked by incubation with 130 µL 1% bovine serum albumin (Sigma-Aldrich) in phosphate-buffered saline (PBS) for 1 hour at 37°C, after which plates were washed twice with PBS supplemented with 0.05% Tween. A 3-fold serial dilution of the heat-inactivated cell suspensions was made in 100 µL PBS supplemented with 0.1% Tween (PBST); 1 µg/mL of Prn and Ptx were used as reference. The suspensions were incubated for 1 hour at 37°C followed by 2 washings. The Prn monoclonal antibody (MAb) (PeM85) that was used binds to the linear epitope GGFGPGGFGP present in the repeat region 1 of all known Prn variants, except Prn13 (*15*). The Ptx MAb (3F10) binds to a conformational epitope in the *PtxA* subunit (*16*). All strains selected for the ELISA experiments produced Prn2 and PtxA1 (Table 1). The MAbs were diluted in PBST, added to the wells, and incubated for 1 hour at 37°C, followed by 2 washings. To detect bound MAbs, plates were incubated with horseradish peroxidase–conjugated polyclonal rabbit anti-mouse IgG (DakoCytmation, Glostrup, Denmark), diluted in PBST, for 1 hour at 37°C, and followed by 2 washings. The optical density at 450 nm was measured with a plate reader (PowerWave HT 340; Biotek, Winooski, VT, USA) and the amount of produced Ptx and Prn were calculated using the KC4 program (Biotek).The ratio of Ptx and Prn production by *ptxP1* and *ptxP3* strains was calculated as follows: Ptx (or Prn) production *ptxP3* strains divided by Ptx (or Prn) production *ptxP1* strains.

### Statistical Analyses

The significance of the increases in illness and death were calculated with the Fisher exact test. Ptx and Prn production was analyzed on the basis of the following considerations: 1) that there are random variations among experiments that influence Ptx and Prn production; 2) that there is a correlation between Ptx and Prn production; and 3) that the distribution Ptx and Prn measurements were skewed. To take into account these considerations regarding sources of random variation, a random intercept model was used and a logarithmic transformation was used before further analysis. Logarithmically transformed Ptx and Prn values were first analyzed with a random intercept model by using SAS PROC MIXED (SAS, Cary, NC, USA) and by using experiment as a random effect. We first tested whether there were differences between *ptxP1* and *ptxP3* strains in the production of Ptx and Prn by analyzing the logarithm of Ptx production and Prn production, respectively, as a dependent variable, and by using experiment as random effect and incubation time (in classes) and type (*ptxP1* or *ptxP3*) as fixed effects. To determine whether the ratio of production in *ptxP3* versus *ptxP1* strains differ significantly for Prn and Ptx, we further fitted a multivariate model with both factors (Ptx and Prn) as dependent variables, again using experiment as random effect, and allowing all variance parameters to be factor (Ptx or Prn) specific. In this model the interaction between *type* (*ptxP1* or *ptxP3*) with factor (Ptx or Prn) then gives the required P value.

## Results

### Polymorphism of the Pertussis Toxin Promoter

**Figure 1:**
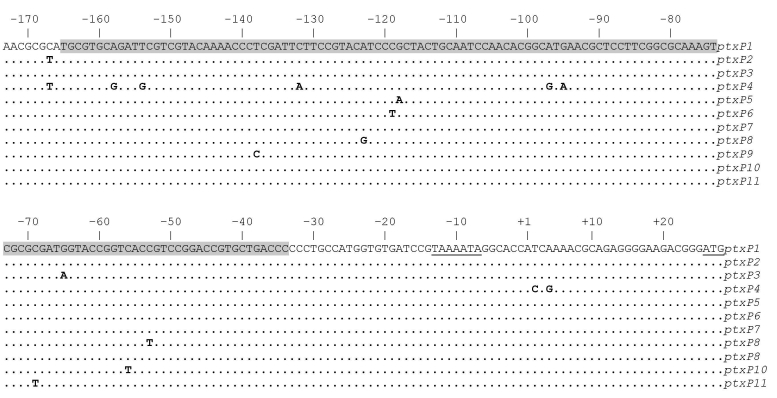
Alleles of pertussis toxin promoter (*ptxP*) observed worldwide. Bases are numbered –173 to +27 relative to the start of transcription (+1). The region to which 6 dimers of BvgA, the global regulator of *B. pertussis* virulence genes, bind is shaded. The –10 sequence motif and initiation codon are underlined. The DNA region –370 to –174, not shown here, was devoid of polymorphism. Locations of transcriptional signals and BvgA bindings sites are based on Bartoloni et al. (*16*).

The synthesis and export of Ptx requires 14 genes, which are co-transcribed from *ptxP* (*17*). *ptxP* comprises a region of ≈170 bases upstream of the Ptx subunit gene *ptxA* and contains the RNA polymerase binding site and 6 binding sites for the BvgA dimer (*18*). BvgA is a global regulator of *B. pertussis* virulence genes, and cooperative binding of BvgA to *ptxP* is required for efficient transcription of ptx (*18*). We investigated polymorphism in *ptxP* by sequencing a DNA region of ≈380 bases upstream of *ptxA* by using a collection of 1,566 *B. pertussis* strains from 12 countries isolated during 1935–2004. Polymorphism was found to be restricted to the DNA region implicated in binding of RNA polymerase and BvgA. Eleven *ptxP* alleles were identified (Figure 1).

### Geographic and Temporal Differences in *ptxP* Frequencies

Next we investigated geographic and temporal differences in *ptxP* frequencies. The following geographic regions were distinguished: the Netherlands, the continents of Africa, Asia, Europe (excluding the Netherlands), North America, and South America. Two periods, chosen on the basis of the appearance of *ptxP3* strains in the Netherlands, were compared: 1935 through 1990 and 1991 through 2004 (Table 2). Only strains from the later period were available from South America. Two *ptxP* alleles were found to predominate worldwide, *ptxP1* and *ptxP3,* and the remaining 9 alleles were pooled. Strains with *ptxP1* predominated in the first period and were found in lower frequencies in the second period (global frequencies were 88% and 47%, respectively). A reverse trend was observed for the *ptxP3* strains (global frequencies, 3% and 52%, respectively). In the first period, *ptxP3* strains were only detected in the Netherlands and the United States (frequencies were 3% and 13%, respectively). The only region in which *ptxP3* strains were not detected was Africa, where only *ptxP1* strains were found. The minor *ptxP* alleles were observed in higher frequencies during 1935–1990 compared with 1991–2004 (global frequencies were 9% and 1%, respectively). The differences in *ptxP* allele frequencies may be due to sampling bias, geographic factors, or differences in vaccines, vaccination history, and vaccination coverage. Nevertheless, these data provide strong evidence that, in most parts of the world, *ptxP3* strains emerged recently and replaced the resident *ptxP1* strains. The *ptxP3* allele was first detected in a strain isolated in the United States in 1984.

**Table 2:** Worldwide frequencies of *ptxP1* and *ptxP3* during 1935–1990 and 1991–2004*

Region†	1935–1990					1991–2004			
	*ptxP1*	*ptxP3*	Other‡	N		*ptxP1*	*ptxP3*	Other‡	N
The Netherlands	89	3	8	265		47	53	0	614
Africa	100	0	0	11		100	0	0	7
Asia	100	0	0	12		83	13	3	30
Europe§	73	0	27	22		46	53	0	577
North America	50	13	38	8		20	80	0	10
South America	–	–	–	0		10	80	10	10
Total	88	3	9	318		47	52	1	1,248

*ptxP, pertussis toxin promoter. Allele frequencies are given in percentages. †The following countries represented the continents: Africa: Senegal; Asia: Japan and Australia; Europe: Denmark, Finland, France, Germany, Italy, Sweden; North America: USA; South America: Argentina. ‡Nine *ptxP* alleles were found in low frequencies: *ptxP2*, *ptxP4*, *ptxP5*, *ptxP6*, *ptxP7*, *ptxP8*, *ptxP9, ptxP10,* and *ptxP11*. §Dutch strains were excluded.

To investigate if *ptxP1* and *ptxP3* alleles were linked to other polymorphisms in *ptx* genes, the gene clusters from 2 *ptxP1* and 2 *ptxP3* strains were sequenced. The *ptx* sequences were identical, except for a single point mutation in *ptxC*. The single nucleotide polymorphism (SNP) in *ptxC* has been described previously, does not result in a change in amino acid sequence, and is therefore most likely selectively neutral (*19*). To study the linkage, *ptxC* was sequenced in 249 *ptxP1* and 142 *ptxP3* strains. Linkage between *ptxP1-ptxC1* and *ptxP3-ptxC2* was 100% and 98%, respectively. Only 3 strains harbored the combination *ptxP3*-*ptxC1*.

### Association of the *ptxP3* Allele with the Resurgence of Pertussis in the Netherlands

**Figure 2:**
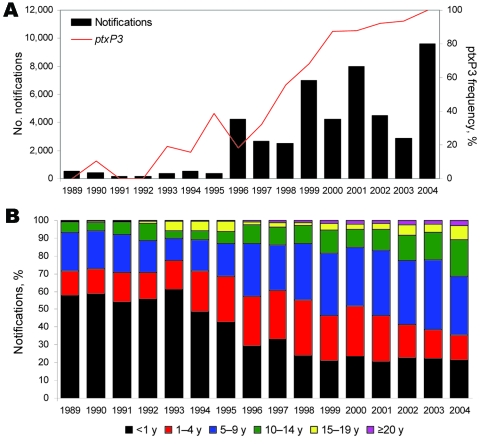
Relationship between the emergence of pertussis toxin promoter 3 (*ptxP3*) strains and the epidemiology of pertussis in the Netherlands, 1989–2004. A) Temporal trends in the frequencies of *ptxP3* strains and notifications. In this period 99% of the strains harbored either *ptxP1* or *ptxP3.* B) Shift in age-specific distribution of notifications.

The availability of a large strain collection allowed us to analyze temporal trends in the Netherlands in more detail. From 1989 through 2004, a total of 99% of the strains harbored *ptxP1* or *ptxP3.* In this period, *ptxP1* was gradually replaced by *ptxP3,* which increased in frequency from 0% in 1989 to 100% in 2004. A close temporal relationship was shown between the increase in *ptxP3* frequency and mandatory pertussis notifications (Figure 2, panel A). Increased notifications were found in all age groups, however, the largest increase was among persons >5 years of age (Figure 2, panel B). The shift toward older age categories coincided with emergence of *ptxP3* strains. There was no change in age distribution from 1989 through 1992, which preceded the emergence of the *ptxP3* allele.

### Ptx and Prn Production by *ptxP1* and *ptxP3* Strains

**Figure 3:**
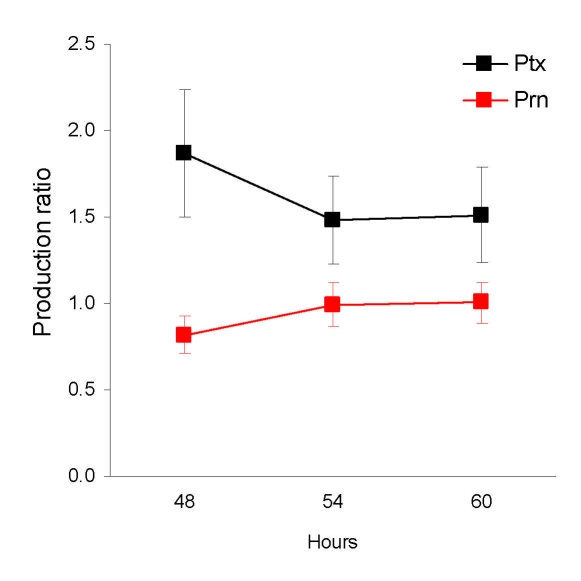
Production of pertussis toxin (Ptx) and pertactin (Prn) by pertussis toxin promoter 1 (*ptxP1*) and *ptxP3* strains. Strains were incubated for the 48, 54, and 60 h, after which the amount of Ptx and Prn was determined by ELISA. The production ratio was calculated as follows: *ptxP3* strain values/*ptxP1* strain values; 8 strains, 4 *ptxP1* strains and 4 *ptxP3* strains, were used. The experiment was performed 3 times. Error bars indicate 95% confidence intervals. The Ptx and Prn ratios were significantly different from 1 (p<0.0001 and 0.03, respectively).

The effect of polymorphism in *ptxP* was assessed by determining the ratio of Ptx produced by *ptxP1* and *ptxP3* strains (Ptx produced by *ptxP3* strain / Ptx produced by *ptxP1* strain) after 48, 54, and 60 h growth on plates. In addition, we assessed the production of a second virulence factor, Prn, which is also regulated by *bvg*. No polymorphism was observed in the Prn promoter of the 8 strains analyzed. Data from 4 *ptxP1* and 4 *ptxP3* strains were pooled (Figure 3). The Prn ratios were slightly lower than 1, indicating that *ptxP3* strains produce slightly less Prn than *ptxP1* strains (average over all time points 0.94; p = 0.03). In contrast, the Ptx ratio was significantly larger than 1 (average over all time points 1.62; p<0.0001), indicating that *ptxP3* strains produce more Ptx than *ptxP1* strains under the growth conditions tested. The Ptx and Prn ratios were significantly different (p<0.0001).

### Evidence for Increased Virulence of *ptxP3* Strains in Humans

Ptx is assumed to contribute to the severity of infection. Therefore, we investigated whether *ptxP3* strains were more virulent in humans than *ptxP1* strains by comparing the incidence of hospitalizations, deaths, and lethality (ratio of deaths to hospitalizations) in the Netherlands during 2 periods, 1981 through 1992 and 1993 through 2004, with low (1.6%) and high (54.5%) *ptxP3* frequencies, respectively (Table 3). All 3 parameters showed a statistically significant increase (1.41, 10.21, and 7.23 times respectively; p values <0.0001, 0.0058, and 0.03, respectively), suggesting that *ptxP3* strains are more virulent in humans.

**Table 3:** Increases in illness and death caused by pertussis in 2 periods with low and high frequencies of *ptxP3* strains in the Netherlands*

Parameter	*ptxP3* frequency, %	Hospitalizations/100,000	Deaths/100,000	Lethality†
1981–1992	1.6	1.38	0.00057	0.00041
1993–2004	54.5	1.95	0.00582	0.00299
Increase	33.1	1.41 (1.34–1.49)	10.21 (1.31–79.11)	7.23 (0.93–56.07)
p value		<0.0001	0.0058	0.03

*ptxP, pertussis toxin promoter. Numbers in parentheses are 95% confidence intervals. †Lethality = no. of deaths / no. of hospitalizations.

## Discussion

The persistence of pertussis in the face of intense vaccination is unexpected because *B. pertussis* is extremely homogeneous (*19*–*21*), implying a limited ability to adapt. However, the Ptx promoter showed a relatively high degree of polymorphism, suggesting that fine tuning of Ptx production has adaptive value. Globally, 11 *ptxP* alleles were found in 1,566 strains, 8 of which occurred in the *B. pertussis* population in the Netherlands. Polymorphism was restricted to a region required for transcription of *ptx*. Silent *ptx* genes are found in the closely related species *B. parapertussis* and *B. bronchiseptica* (*17*). The silencing of *ptx* genes indicates that production of Ptx involves benefits and costs. Thus, production of Ptx is beneficial for the pathogen by suppressing host defenses but also involves metabolic costs and increases the number of immunologic targets. Ptx is a major antigen of *B. pertussis*, and Ptx antibodies are used in diagnosing pertussis cases.

Globally, *ptxP1* and *ptxP3* were the most prevalent *ptxP* alleles. In the Netherlands, during 1989–2004, *ptxP1* was completely replaced by *ptxP3*. The replacement of *ptxP1* strains by *ptxP3* strains in recent times is a global phenomenon because it has been observed in 11 countries representing 4 continents; Asia, Europe, and North and South America. Notably, *ptxP3* strains were not observed in Africa. A broad current distribution of *ptxP3* strains was also suggested by a recent study in which strains from 8 European countries were compared by pulsed-field gel electrophoresis (PFGE). One PFGE profile, BpSR11, predominated in 5 of the 8 European countries (*22*). We have found that in the Dutch population all BpSR11 strains carry the *ptxP3* allele (N = 18).

In the Netherlands, emergence of *ptxP3* strains was associated with increased notifications and a shift in disease prevalence toward older age categories. Changes in diagnostic procedures may have contributed to the latter 2 phenomena (*3*). However, hospitalizations, which are less sensitive to surveillance artifacts, also increased concurrently with the emergence of *ptxP3* strains (Appendix Figure). Furthermore, an extensive analysis of surveillance data confirmed a true increase in the pertussis incidence after 1995 in the Netherlands (*3*). The expansion of *ptxP3* strains was also associated with the resurgence of pertussis in Finland, where a large nationwide epidemic was observed in 2003 (*23*).

The SNP distinguishing the *ptxP1* and *ptxP3* alleles is located in a region involved in binding of BvgA*,* the global regulator of virulence gene expression in *B. pertussis*. We hypothesize that the *ptxP3* allele confers increased binding of BvgA compared to *ptxP1,* resulting in increased toxin production. When compared with *ptxP1* strains, *ptxP3* strains produced 1.62 times more Ptx. In contrast, the production of another *bvg*-regulated virulence factor, Prn, was slightly suppressed in *ptxP3* strains compared with *ptxP1* strains (factor 0.94), indicating that increased Ptx production cannot be explained by a global up-regulation of virulence genes.

The expansion of *ptxP3* strains is remarkable and suggests that *ptxP3* increases strain fitness or is linked to other genetic loci that do so. Although we cannot exclude that other loci are involved in the expansion of *ptxP3* strains, several arguments underline the role of *ptxP3*. First, the high degree of polymorphism in the *ptxP* promoter indicates positive selection. Second, the increased Ptx production observed by *ptxP3* strains provides a rationale for its emergence. It has been well established that Ptx plays a central role in immune suppression. Ptx enhances colonization of naive and immune mice by targeting macrophages and neutrophils (*24*,*25*). Ptx also suppresses antibody responses (*26*). The *ptxP3* allele was found to be associated with 2 *ptxC* alleles, *ptxC1* and *ptxC2,* which are distinguished by a silent SNP. This finding suggests that the *ptxP3* allele is found in different genetic backgrounds, which may be explained by homoplasy or horizontal gene transfer. Both possibilities suggest that *ptxP3* confers increased fitness. In most strains (98%), *ptxP3* was linked to *ptxC2*. Furthermore, genomic profiling of Dutch *B. pertussis* strains indicates that *ptxP3* strains are closely related, and are characterized by a chromosomal deletion (*27*). Thus, it is likely that, in the Netherlands, *ptxP3* strains arose mainly by clonal expansion. We are analyzing a geographically more diverse strain collection to investigate this issue further.

Ptx has been suggested to increase severity of *B. pertussis* infections because the closely related *B. parapertussis,* which does not produce Ptx, generally causes less severe infections (*28*). Furthermore, Ptx causes leukocytosis in humans by inhibiting egression of leukocytes from the vasculature, and high levels of leukocytosis are associated with an increased mortality rate in infants due to pulmonary hypertension (*29*). Thus, the invasion of *ptxP3* strains may result in increased illness and death. Consistent with this assumption, we found that the emergence of *ptxP3* strains in the Netherlands was associated with increased incidence of hospitalizations and deaths and increased lethality. A recent Swedish study also suggested that *B. pertussis* strains differ in virulence. Infection with strains with PFGE profile BpSR11 was associated with a longer duration of hospital stay (*30*). As noted above, BpSR11 strains carry the *ptxP3* allele. An association between Fim2 and increased disease severity was found in a study in the UK (*31*). In contrast, the Swedish study found no association between Fim type and virulence (*30*). Nevertheless, it is conceivable that other polymorphic loci in *B. pertussis* may also affect virulence.

An important issue is whether vaccination has selected for the *ptxP3* strains. Several lines of evidence support this contention. First, *ptxP3* strains were not found in the prevaccination era. Furthermore, although *ptxP3* strains were found in high frequencies in vaccinated populations in the 1990s, they were not detected in Senegal, where vaccination was introduced in 1987 (*32*). Several studies have provided evidence that increased host immunity may select for higher virulence. Vaccination against 2 avian viruses, the Marek disease virus, and the infectious bursal disease virus, were associated with the emergence of more virulent strains (*33*). An important role of host immunity in selecting for virulence is also suggested by the co-evolution of the myxomatosis virus and rabbits (*34*). Furthermore, immune pressure was shown to select for more virulent *Plasmodium chabaudi* parasites in mice (*35*). Based on mathematical modeling, vaccines designed to reduce pathogen growth rate and/or toxicity may result in the evolution of pathogens with higher levels of virulence (*36*).

We propose that the crucial event, which shifted the competitive balance between *ptxP1* and *ptxP3* strains, was the removal by vaccination of immunologically naive infants as the major source for transmission, selecting for strains, which are more efficiently transmitted by primed hosts. Recent studies and historical data indicate an important role of naïve infants in transmission in unvaccinated populations. In a previously unvaccinated population, infant vaccination resulted in a reduction in pertussis in the vaccinated and unvaccinated parts of the population (*37*). Furthermore, in unvaccinated populations, 60%–80% of the pertussis cases were found in children 0–5 years of age, most of whom were probably immunologically naive (*32*,*38*). In most countries infants receive their first vaccination at the age of 2 or 3 months, essentially eliminating transmission by immunologically naive hosts. In primed hosts, increased Ptx production may delay an effective immune response (*24*–*26*) enhancing transmission, and hence, pathogen fitness. Increased Ptx production may also be beneficial for the pathogen because the host requires higher levels of antibodies against Ptx for toxin neutralization. The antigenic divergence observed between vaccine strains and circulating strains (*8*,*9*) may act synergistically with the *ptxP3* polymorphism by enhancing transmission by hosts primed by vaccination. Pertussis among recently vaccinated children is rare, indicating that pathogen adaptation does not play a role unless immunity has waned. Thus, we propose that waning immunity and pathogen adaptation have contributed to the resurgence of pertussis, although other factors such as increased awareness and improved diagnostics have also played a role.

The effect of pathogen adaptation on disease impact may depend on factors such as vaccine coverage and the quality of the vaccine used, which may differ between countries. A relatively weak vaccine used in the Netherlands may have exacerbated the effect of the emergence of *ptxP3* strains on disease impact (*3*). Our results underline the important role of Ptx in the transmission of *B. pertussis* and suggest that an effective way to control pertussis is the improvement of current vaccines to induce Ptx-neutralizing antibodies which persist longer. An important question is whether other childhood vaccines also select for pathogens that are more efficiently transmitted by primed hosts, resulting in increased virulence.

## Supplementary Material

Technical Appendix Netherlands

Appendix Figure Temporal trends in the frequencies of pertussis toxin promoter 3 (ptxP3) strains, notifications, and hospitalizations. In this period, 99% of the strains harbored either ptxP1 or ptxP3. In November 2001, a preschool booster immunization was introduced, which may have reduced hospitalizations.
