# Supplementary figures and images for: Bordetella pertussis Strains with Increased Toxin Production Associated with Pertussis Resurgence
Source: Emerg Infect Dis. 2009 Aug;15(8):1206–13. doi: 10.3201/eid1508.081511 (PMC2815961; doi:10.3201/eid1508.081511)

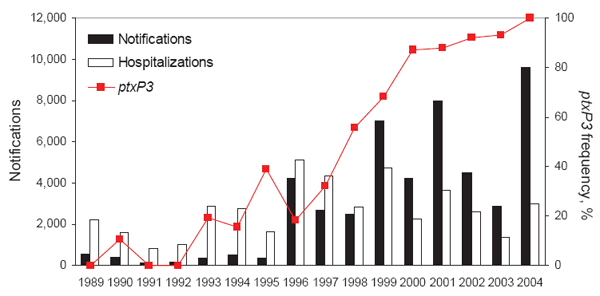

Supplement: Appendix Figure — Temporal trends in the frequencies of pertussis toxin promoter 3 (ptxP3) strains, notifications, and hospitalizations. In this period, 99% of the strains harbored either ptxP1 or ptxP3. In November 2001, a preschool booster immunization was introduced, which may have reduced hospitalizations. [file 08-1511_appF-s2.gif]
